# Proposal of a Global Training Load Measure Predicting Match Performance in an Elite Team Sport

**DOI:** 10.3389/fphys.2017.00930

**Published:** 2017-11-21

**Authors:** Brendan H. Lazarus, Andrew M. Stewart, Kevin M. White, Amber E. Rowell, Alireza Esmaeili, William G. Hopkins, Robert J. Aughey

**Affiliations:** ^1^Institute of Sport, Exercise and Active Living, Victoria University, Melbourne, VIC, Australia; ^2^Collingwood Football Club, Melbourne, VIC, Australia; ^3^Melbourne Victory Football Club, Melbourne, VIC, Australia

**Keywords:** GPS, mixed modeling, monitoring, performance indicators, training load

## Abstract

**Aim:** The use of external and internal load is an important aspect of monitoring systems in team sport. The aim of this study was to validate a novel measure of training load by quantifying the training-performance relationship of elite Australian footballers.

**Methods:** The primary training measure of each of 36 players was weekly load derived from a weighted combination of Global Positioning System (GPS) data and perceived wellness over a 24-week season. Smoothed loads representing an exponentially weighted rolling average were derived with decay time constants of 1.5, 2, 3, and 4 weeks. Differential loads representing rate of change in load were generated in similar fashion. Other derived measures of training included monotony, strain and acute:chronic ratio. Performance was a proprietary score derived from match performance indicators. Effects of a 1 SD within-player change below and above the mean of each training measure were quantified with a quadratic mixed model for each position (defenders, forwards, midfielders, and rucks). Effects were interpreted using standardization and magnitude-based inferences.

**Results:** Performance was generally highest near the mean or ~1 SD below the mean of each training measure, and 1 SD increases in the following measures produced *small* impairments: weekly load (defenders, forwards, and midfielders); 1.5-week smoothed load (midfielders); 4-week differential load (defenders, forwards, and midfielders); and acute:chronic ratio (defenders and forwards). Effects of other measures in other positions were either trivial or unclear.

**Conclusion:** The innovative combination of load was sensitive to performance in this elite Australian football cohort. Periods of high acute load and sustained increases in load impaired match performance. Positional differences should be taken into account for individual training prescription.

## Introduction

Monitoring training is crucial in identifying an athlete's adaptation to a training program and readiness to train/compete, as well as minimizing the risk of non-functional overreaching, injury and illness (Halson, 2014). Training load measures have commonly been used to describe injury risk in team sports (Anderson et al., 2003; Rogalski et al., 2013), however, a paucity of research exists examining the training-performance relationship.

Monitoring of training in sport typically involves multiple measures derived from both internal (Banister et al., 1986; Foster et al., 2001) and external load (Farrow et al., 2008; Boyd et al., 2013), and this can cause a complicated decision making matrix. The more complicated the matrix, the harder it is for practitioners to make informed decisions. In Australian football, in-season training programs include; “on legs” field, resistance, recovery, and cross training sessions plus a match every 6–8 days (Rogalski et al., 2013). In a team sport environment, individual clubs tailor their monitoring systems to suit the emphasis of their training program. For instance, session rating of perceived exertion (RPE) (Scott et al., 2013) or PlayerLoad™ (Boyd et al., 2013) may work for one club but may not be practical for another. The relationship between match performance and a global training load measure in team sport is currently unknown.

The relationship between external and internal load has received increasing attention in the literature. The total distance (TD) and high-intensity distance (HID) covered in matches by soccer players were divided by individualized training impulse (iTRIMP) to provide two ratios; TD:iTRIMP and HID:iTRIMP (Akubat et al., 2014). A *large* correlation (*r* = 0.65–0.69) between the ratios and aerobic fitness indicated the integration from each type of load is beneficial. Further, 62% of the variance in session RPE could be explained by distance, impacts and total body stress (accelerations, decelerations and change of direction) in professional rugby league (Lovell et al., 2013). Indeed, it was reported that playing position impacted the relationship between external and internal load in Australian football, demonstrating that for a given external load, the perceived load may be different due to the interaction between physical capacity and playing position (Gallo et al., 2015). These results support the use of both external and internal factors rather than one measure used in isolation; however, there is no evidence to support the combination of load to examine changes in match performance of elite team sport athletes.

There are a number of derivative measures of internal load that can be used for monitoring and analysis purposes. The effect of training load on performance can be partly considered through the prism of the effect of load on injury risk (Aughey et al., 2015). Two key injury risk factors include excessive accumulations and large changes in load (Rogalski et al., 2013). In Australian football, an increase of at least 1,250 arbitrary units in the previous week's internal load compared to the current week increased the likelihood of in-season injury (Odds ratio = 2.58) (Rogalski et al., 2013). The internal load in this study, calculated with session RPE, encapsulated the weekly periodization including the different training modalities (field, weights, cross training, and running conditioning) and the match. Training monotony (day to day training variability in a given week) and strain (overall stress of the training week) on team sport athletes may also influence match performance (Anderson et al., 2003). In a study involving female collegiate soccer players, 64% of illnesses were associated with monotony and strain, with 53% related to a preceding spike in training load (Putlur et al., 2004). An Australian football team was more successful when training-stress balance calculated using strain was positive (effect size 0.51; ±90% confidence interval 0.41) (Aughey et al., 2015). Training must be carefully managed to avoid residual fatigue and minimize the negative effects of training on injury risk and subsequently match performance. Whilst monotony and strain are valuable monitoring tools, it is not known if they can be applied to measures other than session RPE or weekly rather than daily measures.

The first aim of this study was to quantify load using a unique combination of external and internal variables to provide a global load measure relative to playing position. The second aim was to validate this measure for an elite Australian football team by quantifying the relationship between derived measures of training and match performance.

## Methods

Thirty-six male elite Australian footballers [mean ± standard deviation (SD): age 23.4 ± 3.2 year; height 188.3 ± 8.0 cm; body mass 88.6 ± 8.5 kg], who all played at least one senior match during the 2015 season, participated in this study. Players were all registered to one Australian Football League club, which is the highest level of competition for the sport. In order to obtain a sufficient sample size and to determine whether training measures were affected by position, players were grouped as per their predominant role in the team. The total number of players (*n*) and load observations (*o*) for each position were recorded as follows: defenders (*n* = 13; *o* = 151), forwards (*n* = 13; *o* = 167), midfielders (*n* = 6; *o* = 99), and rucks (*n* = 3; *o* = 27). The study was approved by the Victoria University Human Research Ethics Committee and all players provided informed consent in accordance with the Declaration of Helsinki.

All “on-legs” field training sessions and matches were monitored using Global Positioning System (GPS) units sampling at 10-Hz (MinimaxX, Catapult Innovations, Australia). The device was worn in a custom-made playing uniform, fitting the unit in a pouch, between the scapulae. The validity and reliability (coefficient of variation as a percentage) of GPS units have been established in the literature and is acceptable for TD (1.9%), high velocity running (4.7%), accelerations (4.9%), and decelerations (11.3%) (Varley et al., 2012; Rampinini et al., 2015). Activity profiles were assessed with the following parameters: training and match TD (m), training and match high velocity distance (>5.5 m.s^−1^; m), match average speed (m.min^−1^), match high accelerations (>3 m.s^−2^) and decelerations (<-3 m.s^−2^). During the competitive season, a typical training week comprised of two main “on-legs” field training sessions, two recovery sessions and one competitive match which were all included for the calculation of load. Data were downloaded into proprietary software (Catapult Sprint v5.1.7) and filtered to remove any transition periods (e.g., drinks and quarter breaks), to not underestimate the proportion of high velocity distance or average speed (White and MacFarlane, 2013).

A wellness diary was completed by all players in the morning 2 days post-match to assist in monitoring the recovery process. Categories were chosen based on specific areas of interest to performance staff as previously described (Buchheit et al., 2013). Three main categories titled readiness to train, soft tissue status and overuse/stress risk included 12 items, rated on a 10-point Likert scale ranging from 1 (feeling as bad as possible) to 10 (feeling as good as possible). The individual items were then added together to provide a quantitative score of the overall perceived wellness for each player.

Weekly global load was quantified by combining external load (GPS) variables with internal responses (wellness). Parameters were weighted by importance by performance staff in regards to fatigue inducing load and monitoring susceptibility to injury. The following weightings were applied to each variable; total training distance/100, total match distance/100 × 1.5, training high velocity distance/20, match high velocity distance/10, match work rate/3, match high accelerations × 10, match high decelerations × 20, and wellness diary score × 3. After the weightings were applied, the resulting measure was expressed in arbitrary units. 1-, 3-, and 4-week rolling means were calculated for load.

Various derivative measures of training load were then calculated. The smoothed load is an exponentially weighted rolling average that accounts for the decaying effects of load using a decay factor λ (lambda) (Hunter, 1986). The smoothed load for each week is calculated as λ × (the previous week's cumulative load) + (1 – λ) × (the smoothed load up to that point). The decay factor λ defines a time constant of 1/λ, which represents the period that contains ~2/3 of the total weighting in calculation of the smoothed load. Smoothed loads were generated with λ-values of 0.67, 0.5, 0.33, and 0.25 (time constants of 1.5, 2, 3, and 4 week). Our expression for the time constant of 1/λ is different from the value (2 – λ)/λ suggested recently (Williams et al., 2016). This approach was taken to ensure the smoothed load of a given period has the highest correlation with the simple cumulative load of a similar period (Esmaeili et al., unpublished observations).

A formula similar to that for the smoothed load was used to calculate a predictor variable called differential load, representing the smoothed rate of change in load from one week to the next. In this case, the previous week's load in the above formula was replaced with the change in load between the current and previous week. Differential loads with time constants of 1.5, 2, 3, and 4 week were generated.

Training monotony was calculated by dividing the 3-week rolling mean load by the SD of the 3-week of weekly load. Training strain was calculated by multiplying the monotony by the 3-week rolling mean. It should be noted that our method of calculating monotony and strain is slightly different to the traditional approach due to monitoring weekly global load data rather than daily measures (Foster et al., 2001). A ratio of acute:chronic training was calculated by dividing the 1-week load by the 4-week rolling mean (Hulin et al., 2015).

Performance scores were obtained from Champion Data (Southbank, Australia; http://www.championdata.com.au), the official provider of Australian Football League statistics (Mooney et al., 2011). The scores are based on effective and ineffective skill execution throughout a match (Sullivan et al., 2014).

The effect of each training measure on match performance was modeled with two separate quadratic mixed models in the Statistical Analysis System (version 9.4, SAS Institute, Cary, NC). First, a simpler model was used to understand the extent to which changes in training alone explained any changes in match performance. A within-player SD of the training measure was used to assess the magnitude of the effect of the measure within playing positions; the SD was calculated by taking the square root of the mean of the squares of the players' SD (weighted by the degrees of freedom). Fixed effects in this model were the intercept, the training measure, and the square of the training measure (which together estimate the mean quadratic); the percentage of match-time played was also included as a simple linear effect, because time on field can modify intensity of activity and potentially performance (Mooney et al., 2013). Random effects in the model were player identity (to estimate different between-player means across the season), the interaction of player identity with the training measure and with the square of the training measure (to estimate individual differences in the players' quadratics), and the residual (within-player match to match variability). A more complex model consisting of additional fixed and random effects was then devised to adjust for potential confounders of the apparent training effects: substitution of players during a match (fixed effects for subbed on or off, each coded as a dummy variable), and match identity (a random effect, to adjust for mean differences in performance between matches).

Effects of training on performance were estimated as the change in performance for a typically very low (−2 SD), low (−1 SD), mean, high (+1 SD), and very high (+2 SD) value of the load. Uncertainty in each effect was expressed as 90% confidence limits and as probabilities that the true effect was substantially beyond ±5 raw units, representing the smallest important change; derived by multiplying 0.2 by the observed-between SD averaged across the position groups. Effects were standardized and interpreted using non-clinical magnitude based inferences (Hopkins et al., 2009). Thresholds of clear effects were: <0.2, trivial; 0.2– <0.6, small; 0.6– <1.2, moderate; 1.2– <2.0, large.

We also performed a reliability analysis of the training measures to determine the magnitude of mean differences between players, since substantial differences would complicate the interpretation of the effects of within-player changes in training on performance. True between-player SD's for each training measure were derived from the reliability analyses. Fixed effects were seasonal trend and problems that caused any adjustments to training (injury or illness, defined as a player not participating in full training, coded as a dummy variable). Random effects accounted for player identity, match identity and match identity interacted with problems.

## Results

**Figure 1:**
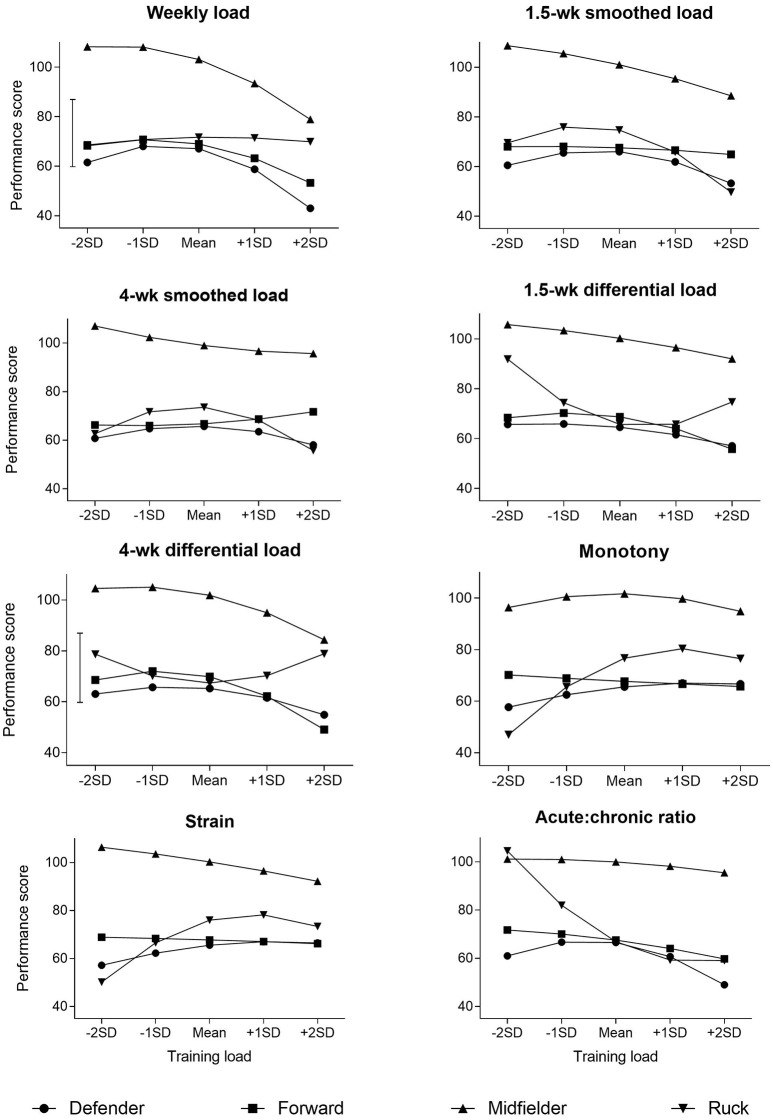
The effect of training measures on performance score. Data are presented as predicted performance scores for the mean training measure and two within-player SD below and above the mean. The capped line represents the observed between-SD averaged over each position, which is used to derive the smallest important change by multiplying by 0.2. The observed between-SD for all training measures and position groups is ~25.

Descriptive statistics for all training measures and performance are summarized in Table 1. The true between-player SD's for each training measures were as follows: weekly load, 34; 1.5-week smoothed, 31; 4-week smoothed, 36, 1.5-week differential 3.8; 4-week differential, 3; monotony, 2.1; strain, 1,562; and acute:chronic ratio, 0.03. However, the values are relatively small, suggesting no substantial differences between players. Effects of the training measures on performance derived from the simple model are presented in Table 2. Recommendations for training are based on the magnitude of the effect of low or high changes (±1 SD) in training on match performance (Table 2). Predicted performance scores by given value of each training measure, two-within player-SD below and above the mean, are displayed in Figure 1.

**Table 1:** Descriptive statistics of performance score and training measures for the playing-position groups. Data presented as mean ± within-player SD.

	**Defender**	**Forward**	**Midfielder**	**Ruck**
Performance score^a^	65 ± 27	68 ± 28	101 ± 27	80 ± 21
Weekly load	541 ± 138	576 ± 144	560 ± 135	475 ± 98
1.5-week smoothed load	528 ± 110	557 ± 120	544 ± 112	459 ± 81
4-week smoothed load	482 ± 85	504 ± 98	495 ± 92	406 ± 62
1.5-week differential load	27 ± 108	38 ± 110	33 ± 101	31 ± 86
4-week differential load	20 ± 39	24 ± 38	22 ± 36	23 ± 26
Strain	5, 334 ± 5, 917	4, 676 ± 4, 665	5, 649 ± 6, 782	4, 070 ± 3, 130
Monotony^b^	9.3 ± 10.8	7.7 ± 7.1	9.5 ± 10.8	8.5 ± 6.3
Acute:chronic ratio	1.04 ± 0.24	1.07 ± 0.24	1.07 ± 0.23	1.10 ± 0.29

a *Performance score, load measures and strain have arbitrary units*.

b *Monotony and acute:chronic ratio are dimensionless*.

**Table 2:** Effects of the training measures on performance score derived from the quadratic mixed model.

	**Change in performance score**^a^ **(mean;** ±**90%CL)**		
	**−1 SD from the mean load**	**+1 SD from the mean load**	**Recommendation for training**
**WEEKLY LOAD**			
Defender	**0.9;** ±**2.9**^000^	–**8.3;** ±6.2^**^	Reduce by 0 to ~1 SD
Forward	**1.7;** ±**2.9**^00^	–**5.8;** ±5.7^*^	Reduce by 0 to ~1 SD
Midfielder	**5.0;** ±**4.2** ^*^	–**9.7;** ±6.8^**^	Reduce by >1 SD
Ruck	−1.0; ±9.3	−0.3; ±10.8	No change
**1.5-WEEK SMOOTHED LOAD**			
Defender	–**0.5;** ±**2.9**^000^	−4.1; ±5.9^*^	No change
Forward	**0.5;** ±**2.8**^000^	−1.1; ±4.9^00^	Reduce by 0 to ~1 SD
Midfielder	**4.4;** ±**4.1** ^*^	–**5.7;** ±5.9^*^	Reduce by >1 SD
Ruck	1.2; ±7.8	−8.7; ±13.3^*^	Reduce by ~1 SD
**4-WEEK SMOOTHED LOAD**			
Defender	–**0.9;** ±**2.9**^000^	−2.3; ±5.1^00^	No change
Forward	–**0.8;** ±**2.8**^000^	1.9; ±4.2^00^	Increase by >1 SD
Midfielder	3.5; ±5.3^*^	−2.2; ±6.4^00^	Reduce by >1 SD
Ruck	−1.9; ±5.5^000^	−5.3; ±10.8	No change
**1.5-WK DIFFERENTIAL LOAD**			
Defender	1.3; ±4.0^00^	–**2.9;** ±**3.8**^00^	Reduce by ~1 SD
Forward	1.5; ±4.1^00^	–**4.8;** ±3.4^*^	Reduce by ~1 SD
Midfielder	3.1; ±6.2^*^	–**3.8;** ±5.1^*^	Reduce by >1 SD
Ruck	8.7; ±10.7^*^	0.1; ±5.3	Reduce by >1 SD
**4-WEEK DIFFERENTIAL LOAD**			
Defender	**0.5;** ±**3.1**^000^	–**3.6;** ±4.4^*^	Reduce by 0 to ~1 SD
Forward	**2.1;** ±**3.0**^00^	–**7.6;** ±4.1^**^	Reduce by ~1 SD
Midfielder	**3.2;** ±**4.6**^00^	–**6.9;** ±5.6^*^	Reduce by ~1 SD
Ruck	2.8; ±6.4^00^	2.9; ±6.1^*^	Reduce by >1 SD
**STRAIN**			
Defender	−3.3; ±7.4^*^	1.4; ±4.8^00^	Increase by ~1 SD
Forward	0.6; ±6.4	−0.8; ±4.0^00^	Reduce by 0 to ~1 SD
Midfielder	3.3; ±13.0	−3.8; ±8.5^*^	Reduce by >1 SD
Ruck	−9.3; ±11.6^*^	2.3; ±5.6^00^	Increase by ~1 SD
**MONOTONY**			
Defender	−3.1; ±7.6^*^	1.4; ±5.0^00^	Increase by ~1 SD
Forward	1.2; ±6.8	−1.0; ±4.0^00^	Reduce by >1 SD
Midfielder	−1.1; ±13.1	−1.9; ±6.9^0^	No change
Ruck	−11.1; ±11.3^**^	3.6; ±5.8^*^	Increase by ~1 SD
**ACUTE:CHRONIC RATIO**			
Defender	0.1; ±3.4^000^	–**5.9;** ±4.9^*^	No change
Forward	**2.6;** ±**3.9**^00^	–**3.4;** ±3.7^*^	Reduce by ~1 SD
Midfielder	1.0; ±4.9^00^	−1.8; ±5.7^00^	Reduce by 0 to ~1 SD
Ruck	15.1; ±19.5^**^	−7.6; ±38.1	Reduce by >1 SD

*±90%CL: 90% confidence limits*.

*Probabilistic inference reflecting a change in the performance score exceeding the smallest important change (±5 units)*.

Likelihood for clear substantial effects:

* *possibly*,

** *likely*,

*** *very likely*,

**** *most likely*.

Likelihood for clear trivial effects:

0 *possibly*,

00 *likely*,

000 *very likely*,

0000 *most likely*.

*Results in bold represent effects clear at the 99% level; all others with superscript clear at 90%*.

a *Performance scores have arbitrary units*.

b *Predicted values refer to Figure 1*.

*The effects are the changes in performance score between the predicted values^b^ with confidence limits with probabilistic inferences, and with recommendations for training for playing-position groups*.

Performance was generally highest near the mean or ~1 SD below the mean of each training measure. However, *small* decrements in performance were observed for the following training measures when increased 1 SD above the mean; weekly load for all positions (except rucks), 1.5-week smoothed for all positions (except forwards), 4-week smoothed for rucks, 1.5-week differential for midfielders and forwards, 4-week differential for all positions and acute:chronic ratio for defenders and forwards. Training monotony and strain produced mainly trivial effects; however, *small* reductions in performance were observed for the following positions when below the mean: rucks (monotony and strain) and midfielders (strain). The effect of 2-, 3-, and 4-week rolling averages were mostly trivial or unclear for all positions (data not shown).

Results from the complex model indicated the relationship between integrated load and match performance was not substantially different when adjusted for additional confounding factors. Due to the high volume of data and in order to avoid duplication, the results from the complex analysis are not shown here. Nevertheless, it is important to note that the effect of training remained causal and was not due to mean changes in training accompanied by mean changes in performance.

## Discussion

The main findings of this study are: (1) a combination of external and internal load was sensitive to changes in performance in this Australian football cohort; (2) performance was typically highest when training measures were at the mean or 1 SD below; (3) Performance was substantially reduced when weekly load (all positions except rucks), 1.5-week smoothed (all positions except forwards) and 4-week differential (all positions) were above the mean; (4) the effects of monotony and strain were mainly trivial; and (5) acute:chronic ratio can also be used as a performance monitoring tool for team sport athletes.

This is the first study to utilize a global training load measure to assess changes in match performance in team sports. We conclude that unique monitoring systems that are specifically designed by performance staff have practical applications in elite Australian football. A system that provides a singular and effective measure of global load can be advantageous to make practical decisions on a weekly basis for individual players. However, care must be taken when comparing training load between studies or an individual team setting and interpreting results, as the effects of load could be contributed by GPS or wellness measures. Despite being a case study, an innovative use of quantifying load and quadratic modeling has been presented here. The results of this study should therefore be treated as promising but preliminary.

When global weekly load was increased above the mean, there were decrements in performance. Australian football is characterized by repeated high-intensity running interspersed with periods of low-intensity activity (Mooney et al., 2013). Players compete in physically demanding matches on a weekly basis which can result in increased muscle damage and fatigue (Mooney et al., 2013). Weekly in-season training periodization typically comprises of 6–8 days break between matches, which includes post-match recovery, strength and skill sessions. As it takes up to 72 h to recover from an Australian football match (Cormack et al., 2008), players may have residual fatigue throughout the remainder of the week, influencing subsequent performance. Practitioners may benefit from implementing a practical test to monitor neuromuscular function, indicative of fatigue, to adjust training loads as required, and improve performance in subsequent matches. Monitoring flight:contraction time of a countermovement jump and comparing pre- to post-match values appears to be the most useful (Cormack et al., 2008). The findings in the current study are in line with a reported decline in match performance in elite Australian footballers who were not sufficiently recovered from the previous match (Hunkin et al., 2014). The performance decrements were explained by increased pre-match creatine kinase concentration 485% greater than base-line (players in a rested state) (Hunkin et al., 2014). Elevated pre-match creatine kinase may represent incomplete or insufficient recovery from the preceding weeks, indicating the presence of chronic muscle damage. Appropriate recovery is crucial to ensure players are ready to physically compete in matches and avoid compromising their performance.

Increases in load over shorter periods of smoothed load (1.5-week) substantially decreased the performance of the midfield group. A key role of the midfielders is to be involved with both attack and defense (McLeod and Jaques, 2006). Midfield players have greater physical requirements as they complete a higher volume of running during matches and training (Wisbey et al., 2010). Our finding is supported by 1 week of increased training load that resulted in greater muscle damage and reduced running performance (decreased peak sprint velocity and TD covered) during Australian football match simulation (Slattery et al., 2012). A non-motorized treadmill protocol was used to replicate the sport-specific activity profile of Australian football match-play (Sirotic and Coutts, 2007). The period of heavier internal load was sufficient to increase markers of muscle damage, reduce energy production via glycolytic pathways and impair performance (Slattery et al., 2012). It is important to note that the effect of an increase in 4-week smoothed load above the mean was trivial. It appears the fitness acquired from higher loads exceeds the fatigue that induces it (Bingham, 2015). Therefore, it is likely the midfield group improved their stress tolerance to extended bouts of accumulated load which minimized the effect on match performance.

To our knowledge, this is the first study to analyse the rate of change in load and match performance in a team sport. Performance was reduced in all positions after an increase in 4-week differential load. In a study involving tennis players, a 4-week overloading training period evoked higher symptoms of perceptual stress, which reflected a decline in the athlete's ability to cope with the training stimulus (Gomes et al., 2013). Further, running performance and V°O_2max_ were reduced by 9.2 ± 7.7% and 4 ml^.^kg^−1^min^−1^, respectively following 6 weeks of intensified training in professional rugby players (Coutts et al., 2007). The reduction in performance suggests the training program induced non-functional overreaching, and was above the tolerance of improving fitness. Team sport athletes that experience sustained increases in week to week load without sufficient recovery may benefit from a deload period, which elicits improvements in performance and reductions in muscle damage (Coutts et al., 2007).

In this Australian football team, changes in monotony and strain did not affect the training-performance relationship. An explanation is our calculation of monotony and strain that was derived differently to the method which is traditionally used in the literature (Foster et al., 2001; Anderson et al., 2003; Aughey et al., 2015). Whilst the monotony and strain data in this study may be difficult to interpret, it should not deter practitioners from using such measures. Training monotony and strain, calculated with session RPE, have substantial relationships with match outcome in elite Australian football (Aughey et al., 2015) and injury and illness in women's basketballers (Anderson et al., 2003). The efficacy of monotony and strain derived in this way remains unknown, which warrants further research into the adaptability of such measures to the training load methodology like the one used in this study.

The acute:chronic ratio is commonly used to monitor if an athlete's acute workload is more or less than what the athlete is prepared for during the chronic period (Hulin et al., 2015). The acute:chronic ratio has largely been applied for predicting injury risk (Hulin et al., 2015; Gabbett, 2016), but it has yet to be used as a predictor of performance. In terms of injury risk, an acute:chronic ratio between 0.8 and 1.3 is identified as the “sweet spot,” while ratios ≥1.5 represent the “danger zone” (Gabbett, 2016). For comparison, in this case study across all positions, the mean ratio was ~1.0, which equates to the sweet spot for injury risk. While the effects were mainly *trivial*, performance was generally higher near or below the mean, which suggests the “sweet spot” for maximizing performance is similar to injury risk. It is likely that different sports, teams, and load monitoring systems will have different training-performance relationships; therefore, these recommendations should be taken with caution and practitioners are urged to use a framework like that presented here to determine the ideal load in their own cohort of athletes.

## Conclusion

This study reinforces the importance of a load monitoring system in elite sporting environments. Due to the complex nature of training, quadratic modeling appears valuable when examining the training-performance relationship. Coaching and performance staff should avoid prescribing substantially high weekly and sustained increases in load during the competitive period of the season. Positional differences should be taken into account when planning and prescribing training loads across an entire Australian football season.

## Author contributions

Conceived and designed the study: BL, RA, AS, and WH. Analyzed the data: WH, BL, AE, and AR. Interpreted the results: BL, WH, RA, AS, and KW. Drafted the manuscript and prepared the tables/figures: BL. Edited, critically revised the manuscript, and approved the final version: BL, RA, AS, WH, KW, AE, and AR.

### Conflict of interest statement

The authors declare that the research was conducted in the absence of any commercial or financial relationships that could be construed as a potential conflict of interest.
